# Cognitive correlates of mixed depression

**DOI:** 10.1192/j.eurpsy.2022.261

**Published:** 2022-09-01

**Authors:** A. Simonetti, E. Bernardi, G. Bartolucci, O. Ferrara, S. Ruggiero, M. Di Nicola, D. Janiri, G. Sani

**Affiliations:** Fondazione Policlinico Universitario Agostino Gemelli IRCCS, Department Of Psychiatry, Rome, Italy

**Keywords:** affective disorders, Mixed depression, Neuropsychology, cognitive function

## Abstract

**Introduction:**

Mixed depressive states portend greater rates of impulsivity, attempted suicide, treatment resistance, and poorer outcome than non-mixed forms of depression. The neurocognitive bases of such affective states have not been defined yet.

**Objectives:**

This work represents an attempt to clarify the neuropsychology underlying mixed depressive states.

**Methods:**

Thirty subjects with affective disorders with mixed depression (MxD), 54 subjects with non-mixed depression (nonMxD), 73 euthymic subjects (Eu) and 93 healthy comparisons (HC) underwent a neurocognitive battery including the Trail-Making Test (TMT), the Controlled Word Fluency Test (WFT) and the Semantic Fluency Test (SFT), the Wisconsin Card Sorting Test (WCST, the Rey Auditory Verbal Learning Test RAVLT, the Rey-Osterrieth Complex Figure Test ROCFT, the Raven’s Progressive Matrices (RPM), and the Interference Component of the Stroop Test (ST). Between-group differences were performed through multiple one-way analyses of variance. Post-hoc analyses were performed using Tukey post-hoc tests.

**Results:**

HC performed better than the three patient groups in all the aforementioned neurocognitive tests. Eu performed better in RPM, TMT, SFT than nonMxD, and better on ST WCST than both nonMxD and MxD. MxD showed better performances in RPM, TMT-A, WCST than nonMxD, and more errors and less reaction times in the ST than nonMxD.

**Conclusions:**

Mixed depressive states are characterized by enhanced attentional resources and greater set shifting abilities than non-mixed depressive states. On the other hand, they have less cognitive control than non-mixed depression. Such findings might explain some typical features observed in subjects with mixed depression, such impulsivity, suicidality, emotional reactivity and behavioral dyscontrol.

**Disclosure:**

No significant relationships.

